# Animal experimental research design in critical care

**DOI:** 10.1186/s12874-018-0526-6

**Published:** 2018-07-05

**Authors:** Justin S. Merkow, Janine M. Hoerauf, Angela F. Moss, Jason Brainard, Lena M. Mayes, Ana Fernandez-Bustamante, Susan K. Mikulich-Gilbertson, Karsten Bartels

**Affiliations:** 1 Department of Anesthesiology, Medicine, and Surgery, University of Colorado, School of Medicine, Anschutz Medical Campus, 12401 E. 17th Ave., Leprino Office Building, 7th Floor, MS B-113, Aurora, CO 80045 USA; 2 Adult and Child Center for Health Outcomes and Delivery Science, University of Colorado, School of Medicine, Aurora, Colorado USA; 3 Department of Psychiatry, University of Colorado, School of Medicine, Aurora, Colorado USA; 4 Department of Biostatistics & Informatics, University of Colorado, School of Public Health, Aurora, Colorado USA

**Keywords:** Critical care, Research, Experiment, Study design, Methods

## Abstract

**Background:**

Limited translational success in critical care medicine is thought to be in part due to inadequate methodology, study design, and reporting in preclinical studies. The purpose of this study was to compare reporting of core features of experimental rigor: blinding, randomization, and power calculations in critical care medicine animal experimental research. We hypothesized that these study design characteristics were more frequently reported in 2015 versus 2005.

**Methods:**

We performed an observational bibliometric study to grade manuscripts on blinding, randomization, and power calculations. Chi-square tests and logistic regression were used for analysis. Inter-rater agreement was assessed using kappa and Gwet’s AC1.

**Results:**

A total of 825 articles from seven journals were included. In 2005, power estimations were reported in 2%, randomization in 35%, and blinding in 20% (*n* = 482). In 2015, these metrics were included in 9, 47, and 36% of articles (*n* = 343). The increase in proportion for the metrics tested was statistically significant (*p* < 0.001, *p* = 0.002, and *p* < 0.001).

**Conclusions:**

Only a minority of published manuscripts in critical care medicine journals reported on recommended study design steps to increase rigor. Routine justification for the presence or absence of blinding, randomization, and power calculations should be considered to better enable readers to assess potential sources of bias.

## Background

Despite a significant increase in the volume of biomedical research over the past decade, there has been limited translational success in clinical medicine [1, 2]. Reproducibility specifically for animal research is low [3–5]. In attempts to address this problem, the Animal Research: Reporting of In Vivo Experiments (ARRIVE) guidelines as well as the revised National Institutes of Health grant application process have proposed standards for research involving animals to enhance the quality of experimental design, study conduct, and analysis of results [6–8]. These steps are intended to reduce bias and ultimately improve reproducibility and facilitate the translation of biomedical research to novel clinical applications that improve patient outcomes. Additionally, there is an ethical dilemma regarding animal welfare as well as financial waste related to permitting investment into research without tangible returns [9]. Specifically for the field of critical care medicine, small studies have shown that animal research methodology, study design, and reporting tends to lack rigor in several important areas [10–13].

Improvements in reporting of key experimental design features could enable readers to better judge sources of bias and eventually enhance validity and likelihood of translation. The objective of our study was to evaluate all critical care journals and compare reported animal experimental research in 2005 vs. 2015 regarding power analysis, randomization, and blinding procedures. Our hypothesis was that there had been increased implementation of these methods in 2015 compared to 2005. Also, we sought to provide information on the *status quo* of reported experimental design features to promote rigor.

## Methods

**Fig. 1:**
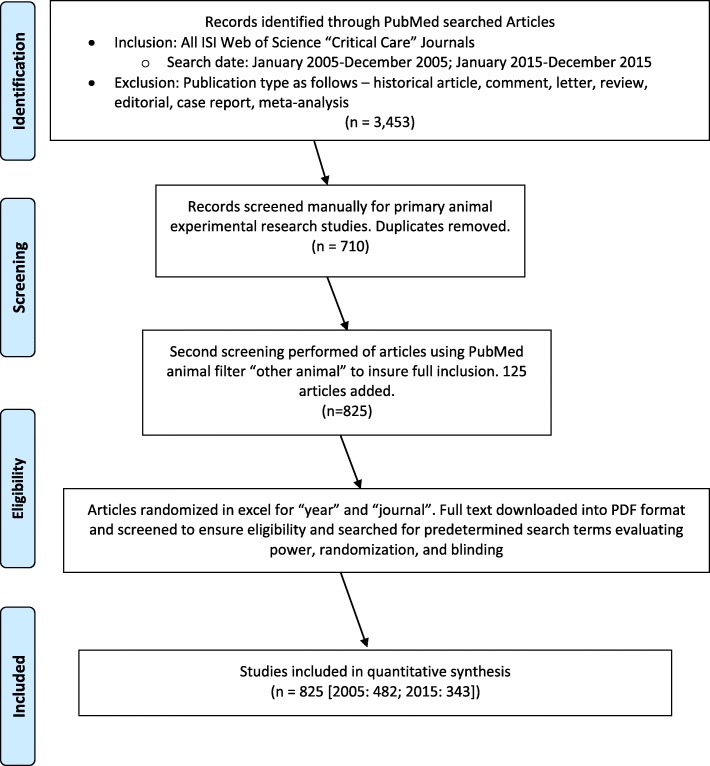
Study flow diagram

We performed an observational bibliometric analysis of animal research published in critical care medicine journals using PRISMA and STROBE guidelines [14, 15]. Journals were selected based on their inclusion on the Thomson Reuters™ *Journal Citation Reports®* subject category “Critical Care Medicine” [16]. A PubMed search included animal experimental studies published in 2005 and 2015. Our primary search criterion was that the article was reporting on an animal study based on an experiment. Animals were further defined as: “any of a kingdom of living things composed of many cells typically differing from plants in capacity for active movement, in rapid response to stimulation, in being unable to carry photosynthesis, and lack of cellulose cell walls” [17]. We excluded meta-analyses, case reports, historical articles, letters, review articles, and editorials. One investigator manually assessed the PubMed search results for animal experimental studies. Then, the PubMed filter “other animals” was applied to the initial search results to detect any animal experimental studies not found in the manual search. Journals that did not publish at least ten animal studies in both 2005 and 2015 were excluded from the analysis (Fig. 1). To assess consistency in the identification of manuscripts reporting on animal experimental research, a second investigator blinded to the results of the first investigator independently searched two journals that were randomly selected from the seven journals included in this study.

Next, we rated all animal studies selected. A computer-generated randomization scheme was used to randomize articles by both year and journal before the analysis (Excel, Microsoft Co., Redmond, WA). Studies were analyzed using their full-text Portable Document Format (PDF). Reporting of power analysis, randomization, and blinding was then graded using a 0–3 point scale (0-not mentioned, 1-mentioned but specified as not performed, 2- performed but no details given, 3-performed and details given) [18]. To assess inter-rater agreement for criterion ratings, we randomly selected 10 % of the total articles for re-rating by a second investigator blinded to the results of the first investigator.

### Statistical analysis

To address the primary hypothesis, ordinal scale rating scores were collapsed into binary (performed/not performed) variables. Chi-square tests were used to examine overall trends in reporting of quality metrics for 2005 and 2015. Simple logistic regression with time as a continuous covariate was used to estimate the effect of time on quality metrics performed and reported in published articles. The reference group was “not performed”, and odds ratios were calculated for the entire 10-year increment in time.

To assess the relationship between year of study and degree of reporting of quality metrics (as ordinal variables), the Wilcoxon Rank Sum test was used. Proportional odds models for ordinal logistic regression was used to calculate an odds ratio for the increase in reporting of metrics in 2015 compared to 2005. The proportional odds assumptions were verified by the Score Test.

Inter-rater agreement was assessed for each of the three metrics (power, randomization, and blinding) using the Cohen’s Kappa and Gwet’s AC1 [19]. Gwet’s AC1 is an alternative inter-rater reliability coefficient to Cohen’s kappa that is more stable in the presence of high prevalence and unbalanced marginal probability [19, 20]. Inter-rater agreement for identification of animal study articles was assessed using the kappa coefficient. The level of agreement was interpreted using the scale for interpretation of Kappa [21]. The statistical analysis was done in SAS 9.4 (SAS Institute, Cary, NC). Statistical tests were performed adjusting for multiple comparisons using the Bonferroni method to maintain an overall 0.05 level of significance.

### Power analysis

For the power analysis, we assumed a 12% absolute increase in reporting incidences for each of the three metrics over a 10-year interval in two independent proportions [18]. We anticipated a baseline reporting level of 5% in 2005 and a reporting level of 17% in 2015. A total of 141 studies in each year (282 total) would yield 80% power to detect an absolute difference in the proportion of metrics identified of at least 12% as significant.

For the randomization metric, we assumed a 13% absolute increase in reporting incidences for each of the three metrics over a 10-year interval in two independent proportions [18]. We anticipated a baseline reporting level of 41% in 2005 and a reporting level of 54% in 2015. A total of 307 studies in each year (614 total) would yield 80% power to detect an absolute difference in the proportion of metrics identified of at least 13% as significant.

For the blinding metric, we assumed a 21% absolute increase in reporting incidences for each of the three metrics over a 10-year interval in two independent proportions [18]. We anticipated a baseline reporting level of 26% in 2005 and a reporting level of 47% in 2015. A total of 109 studies in each year (218 total) would yield 80% power to detect an absolute difference in the proportion of metrics identified of at least 12% as significant.

All power calculations were done using G*Power, version 3.1.9.2. To maintain a 0.05 significance level across the three outcome metrics, the Bonferroni method for multiple comparisons was used to adjust the alpha to 0.017.

## Results

**Fig. 2:**
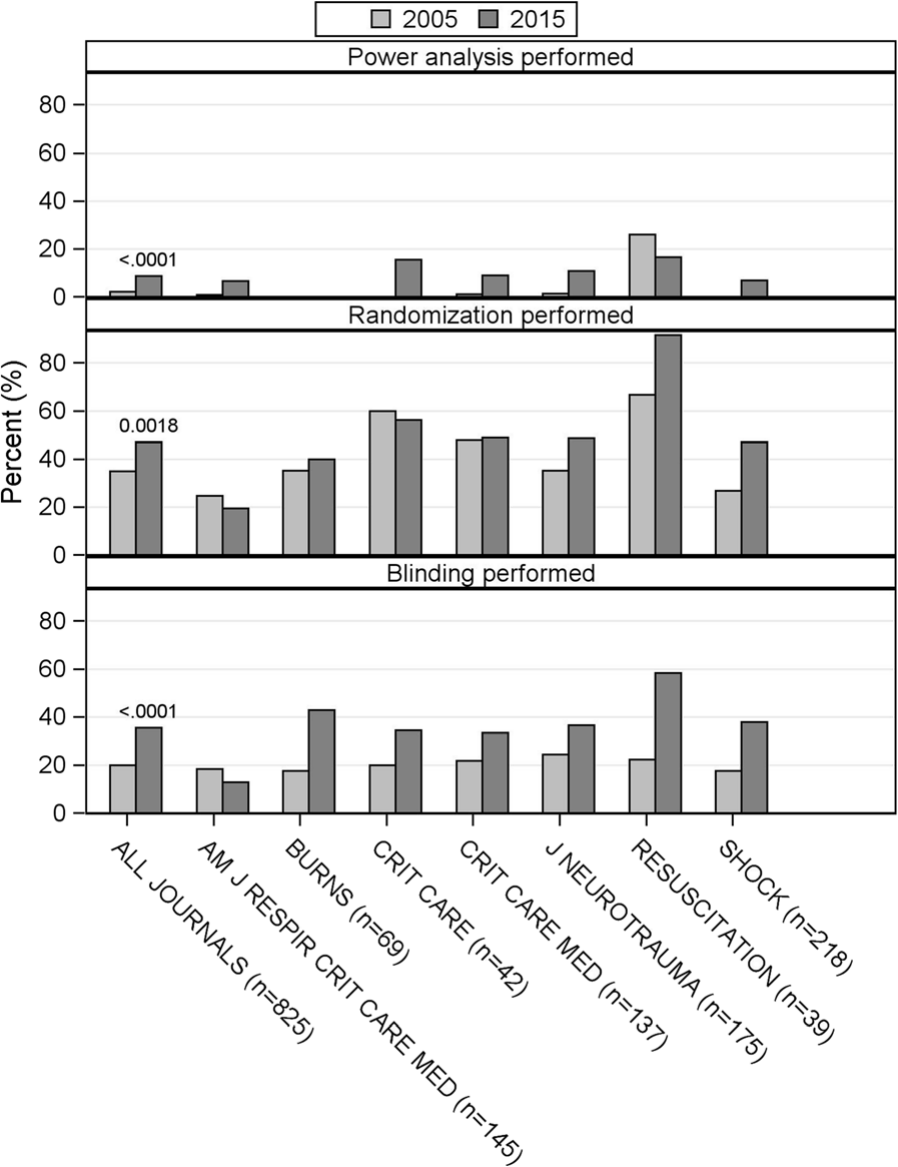
Frequencies of recommended study design feature reporting per journal. Comparison was made using Chi square test

After excluding critical care journals that did not publish at least ten animal studies in each year, seven journals comprising 825 articles (482 in 2005, 343 in 2015) were included in the analysis. They included: American Journal of Respiratory and Critical Care Medicine, Burns, Critical Care, Critical Care Medicine, Journal of Neurotrauma, Resuscitation, and Shock. The odds of any of the three metrics being performed in 2015 were higher than in 2005. The breakdown of the changes in reporting frequencies for each journal is depicted in Fig. 2. For power analysis, the odds were 4.52 times (1.86,11.0) higher, for randomization 1.64 times (1.16,2.31) higher, and for blinding 2.22 times (1.51,3.25) higher in 2015 compared to 2005 (Table 1).

**Table 1:** Reporting of recommended study design features in critical care medicine manuscripts 2005 and 2015 (binary ratings). Comparisons were made using Chi square tests (*P*-value) and simple logistic regression (odds ratio)

Study design feature	Total (*n* = 825)	2005 (*n* = 482)	2015 (*n* = 343)	Bonferroni-adjusted *P*-value	Odds Ratio (98.3%CI)
Power analysis performed	40 (5)	10 (2)	30 (9)	< 0.0001	4.52 (1.86,11.0)
Randomization performed	330 (40)	169 (35)	161 (47)	0.0018	1.64 (1.16,2.31)
Blinding performed	218 (26)	96 (20)	122 (36)	< 0.0001	2.22 (1.51,3.25)

The highest rating of “performed and details given” was present in 2005 vs. 2015 for power analysis in 2% vs. 8%, for randomization in 3% vs. 8%, and for blinding in 7% vs. 13% of manuscripts. An article published in 2015 was 3.26 (1.61,6.61) times more likely to have a higher level of reporting of power analyses than in 2005. 2015 articles were 1.67 (1.21,2.32) times more likely to have a higher level of reporting of randomization than in 2005, and the odds of a higher level of reporting of blinding was 2.10 (1.45,3.04) times greater in 2015 compared to 2005 (Table 2).

**Table 2:** Reporting of recommended study design features in critical care medicine manuscripts 2005 and 2015 (ordinal ratings). Comparisons were made between 2005 and 2015 using Wilcoxon Rank Sum test (P-value) and proportional odds models were used to calculate odds

Study design feature	Total (*n* = 825)	2005 (*n* = 482)	2015 (*n* = 343)	Bonferroni-adjusted *P*-value	Odds Ratio (98.3%CI)
Power analysis					
Not mentioned	769 (93)	464 (96)	305 (89)	< 0.0001	3.26 (1.61,6.61)
Mentioned but not performed	16 (2)	8 (2)	8 (2)		
Performed but no details given	6 (1)	2 (0)	4 (1)		
Performed and details given	34 (4)	8 (2)	26 (8)		
Randomization					
Not mentioned	445 (54)	283 (59)	162 (47)	0.0005	1.67 (1.21,2.32)
Mentioned but not performed	50 (6)	30 (6)	20 (6)		
Performed but no details given	290 (35)	155 (32)	135 (39)		
Performed and details given	40 (5)	14 (3)	26 (8)		
Blinding					
Not mentioned	596 (72)	378 (78)	218 (64)	< 0.0001	2.10 (1.45,3.04)
Mentioned but not performed	11 (1)	8 (2)	3 (1)		
Performed but no details given	143 (17)	64 (13)	79 (23)		
Performed and details given	75 (9)	32 (7)	43 (13)		

For the binary ratings, observed agreement between the two investigators for the 82 articles assessed was 0.95, 0.93, and 0.90 for power, randomization, and blinding respectively. Cohen’s Kappa values indicated moderate agreement for power, almost perfect agreement for randomization, and substantial agreement for blinding. Gwet’s AC1 values indicated almost perfect agreement beyond that which occurs by chance alone (Table 3). Observed agreement between the two investigators in identifying all articles reporting animal experimental research from two randomly selected journals for inclusion/exclusion in this study was 0.99. The kappa coefficient indicates almost perfect agreement beyond that which occurs by chance alone (0.97 (95% CI 0.94,0.99)).

**Table 3:** Inter-rater agreement for binary ratings of metrics using Cohen’s Kappa, Gwet’s AC1, and observed agreement

Study design feature	Cohen’s Kappa	98.3% CI	Gwet’s AC1 Coefficient	98.3% CI	Observed agreement
Power analysis	0.58	0.13, 1.00	0.94	0.88, 1.00	0.95
Randomization	0.85	0.72, 0.99	0.85	0.72, 0.99	0.93
Blinding	0.79	0.62, 0.95	0.82	0.67, 0.97	0.90

## Discussion

The quality of research and reporting of animal studies in critical care medicine journals is an area of increased interest, especially as reproducibility and successful translation of basic science results to clinical application has been low [22–24]. In addition to impeding progress in the development of novel therapies, these issues also present ethical concerns [9, 25–27]. In attempts to improve animal research quality, initiatives such as the ARRIVE guidelines have been created to improve the methodological rigor and to enhance translation [8]. To date, there are few studies examining the reporting of recommended experimental design feature to increase scientific rigor and reduce bias in animal experimental critical care research.

In our study, we evaluated the methodological quality of animal research in critical care journals in 2005 and 2015 and found a significant increase in the reporting of power analyses, randomization, and sample size calculations. Our hypothesis that these metrics are more commonly reported in 2015 compared to 2005 was confirmed. Introduced in 2010, the ARRIVE guidelines [8] may have been one of several factors that led to the improved reporting of recommended study design features in 2015. Our analysis using an ordinal scoring system still found the lowest rating category to be the most common one for every criterion assessed, even in 2015. Contemporary research in the field of critical care reports on recommended procedures to improve experimental design rigor only in a minority of manuscripts. This is in line with the limited published literature on this topic. Bara et al. [13], reviewed 77 animal research articles published in critical care journals over a six-month period in 2012. They found that 61% reported randomization and 6% of these reported some type of allocation concealment and only 2% reported a method of randomization.

Huet et al. [12] highlighted the importance on enhancing animal research quality including improving the use of the 3Rs (replacement, reduction, refinement), which are the guiding principles for ethical animal testing [28–30]. They emphasized, however, that there continues to be poor adherence to these recommendations. Festing et al. [3], emphasized the historical significance of animal research and the major contributions resulting from it: animal research has led to the advancement of immunization medicine, use of vitamins in almost eliminating diseases such as scurvy and rickets, and the discovery of insulin and its effect on metabolic diseases. Yet, they also identified a lack of adherence to good practices of research design as a major impediment to progress in medicine.

Although enhanced translation is the ultimate goal of measures to improve experimental design rigor, it remains to be determined if there has been an improvement in reproducibility or successful translation of animal experimental research results. Given the significant time lag between the description of basic science results and publication of clinical trial results, proof of a direct relationship between reported experimental design rigor and translation to novel therapies for critically ill patients will be challenging. It is also possible that some articles may not have described quality metrics that were in fact utilized in the research protocol. In addition, editors and reviewers may have recommended reporting according to the more recent ARRIVE [8] guidelines during the review process. The observed difference between 2005 and 2015 may, therefore, reflect more a change in reporting as opposed to a change in experimental practices. Of note, an innovative online tool, the “Experimental Design Assistant” was introduced in October 2015 as a guide for researchers to assist in the rigorous design of experiments [31]. However, none of the articles included in our study mentioned utilizing this resource. Further, our search strategy may not have detected all animal research articles in critical care journals in the two time periods examined. However, almost perfect agreement existed between two independent investigators in this regard. Critical care relevant research is published in other (non-critical care medicine specific) journals, and we did not include non-critical care journals in this study. Indeed, when comparing 2005 to 2015, the annual number of animal experimental manuscripts published in critical care journals decreased by 139 articles. This contrasts with findings that overall, publications in the medical literature have been increasing in the last decade [32, 33]. Finally, publication bias was not assessed in this study. Publication bias likely has a significant impact on the quality of animal research and its ability to be translated into successful clinical trials [34, 35].

## Conclusions

The application and reporting of recommended quality metrics in animal experimental research published in critical care medicine journals continue to be modest. However, the increase in reported measures aimed to improve experimental design quality and reduce sources of bias in 2015 compared to 2005 is promising. Reporting of blinding, randomization, and sample size estimates should be encouraged in future animal experimental publications in critical care medicine. The routine justification for the presence or absence of these study design features should be considered in reports on animal experimental research.

## Abbreviations

ARRIVE: Animal Research: Reporting of In Vivo Experiments guidelines
NIH: National Institutes of Health
